# Effects of Mindfulness-Based Stress Reduction (MBSR) on Stress, Heart Rate Variability, Affect, and Wellbeing among People with Schizophrenia

**DOI:** 10.3390/ijerph182211871

**Published:** 2021-11-12

**Authors:** Ae Sil Kim, Mi Heui Jang, Min Jung Sun

**Affiliations:** 1 Department of Nursing, Yeungnam University College, Daegu 42415, Korea; interkim2@hanmail.net; 2 College of Nursing Science, Kyung Hee University, Seoul 02447, Korea; dnfntk0213@khu.ac.kr

**Keywords:** schizophrenia, stress, mindfulness, affect

## Abstract

Mindfulness-based stress reduction programs have been found to be effective in reducing the stress response and improving the psychological wellbeing of various populations. We aimed to confirm the effects of a mindfulness-based stress reduction program on perceived stress, heart rate variability, positive and negative affect, and subjective wellbeing of community-dwelling people with schizophrenia. The participants in this study were 26 people with schizophrenia (experimental group: 14, control group: 12) enrolled in two community mental health centers located in Gyeonggi Province in South Korea. In the experimental group, the mindfulness-based stress reduction program was applied once a week for 60 min over 8 weeks. The experimental group showed a significantly greater decrease in perceived stress and negative affect, as well as significantly greater improvement in heart rate variability than the control group. The mindfulness-based stress reduction program was an effective nursing intervention to reduce stress and negative affect in people with schizophrenia.

## 1. Introduction

The international lifetime prevalence of schizophrenia among noninstitutionalized persons is 0.3% to 0.7% [1]. Schizophrenia is a disease with a high socioeconomic burden due to functional deterioration and chronic progression, to the point that it comprises 20% of all mental health-related direct costs and belongs to the top 10 diseases with the highest disability rate [2]. People with schizophrenia are vulnerable to stress due to difficulties in interpersonal relationships and low self-esteem [3]. For this reason, there is a vicious cycle of being discharged from the hospital and then subjected to rehospitalization due to relapse induced by vulnerability to stress and environmental stressors, resulting in numerous difficulties in returning to society and adapting thereto [4,5].

The personal and internal stress factors experienced by people with schizophrenia in the community include side-effects caused by antipsychotic drugs, other health problems [6], psychotic symptoms such as hallucinations and delusions of persecution, and reduced quality of life and wellbeing due to frequent cycles of hospitalization and discharge [7]. Furthermore, people with schizophrenia experience discrimination in their daily life, as well as a high level of stress from social prejudice and stigma [8]. Psychosocial rehabilitation therapy and drug therapy have been provided to people with schizophrenia to reduce their stress, control symptoms, and restore function [9]. The various approaches to psychosocial interventions that have been developed to date provide significant benefits in improving patients’ symptoms, drug compliance, and relapse [10]. However, most current approaches to psychosocial interventions pay relatively little attention to patients’ acceptance of schizophrenia and their incomprehensible and stressful experiences of psychotic symptoms. Many of them also involve limited strategies to empower self-management of the illness [10].

Recent studies have revealed that mindfulness-based interventions (MBIs) are effective in the treatment of various mental health disorders, and they may improve psychological symptoms and reduce stress in persons with a mental illness [11,12]. Moreover, studies have shown that MBIs are effective at reducing cardiovascular disease, depression [13,14], anxiety [15], and stress [16,17], as well as improving mindfulness, positive affect, negative affect, emotion regulation, wellbeing [18], and quality of life [19]. Mindfulness can be understood as a specific form of meditation that seeks to augment various psychological functions by means of a synergic effort between attention regulation, self-awareness, and emotion regulation, thereby increasing psychological resilience and self-regulation [20]. As an example of an MBI, mindfulness-based stress reduction (MBSR) programs were developed in a behavioral medicine setting for populations with a wide range of chronic pain and stress-related disorders [21]. Scientific evidence has demonstrated that MBSR programs can have a profound benefit via the mind–body connection; specifically, the practice of mindfulness results in an increase of awareness, as individuals purposefully pay attention to the present moment and nonjudgmentally monitor the unfolding of experiences moment by moment [22,23].

Recent systematic reviews and meta-analyses of randomized controlled trials have found that MBIs reduced psychotic symptoms, positive symptoms, negative symptoms, depressive symptoms, and duration of rehospitalization among people with schizophrenia, as well as improving their level of function and awareness of illness [24,25]. In the last few decades, MBIs have rapidly become more popular, and extensive research has explored their efficacy as treatments for various psychological disorders [26]. However, there is limited evidence regarding whether MBIs are applicable for schizophrenia in South Korea. One of the reasons for the scarcity of evidence is that meditation in MBIs was previously considered inappropriate for people with schizophrenia since it might influence their psychotic symptoms [27]. However, in South Korea, studies with conflicting results have been reported. On one hand, a study argued that MBIs are inappropriate for people with schizophrenia because meditation could affect their psychotic symptoms [27]. On the other hand, studies [28,29] have shown that MBIs have a positive effect on depression, emotion, and responses to stress in people with schizophrenia. In light of these discrepancies in the literature, there is a need for an evidence-based study of an MBSR program focusing on stress alleviation with confirmation of the intervention’s effects using objective measures, including physiological indices related to the stress response.

Thus, considering previous studies, this study aimed to apply an MBSR program to people with schizophrenia residing in the local community and to verify its effects. It was hypothesized that engaging in the MBSR program would improve indices of wellbeing and affect, as well as improve stress reactivity among outpatients with chronic schizophrenia. Specifically, the aim of this study was to confirm the effects of the MBSR program on perceived stress, heart rate variability (HRV), positive and negative affect, and subjective wellbeing of community-dwelling people with schizophrenia. The study hypotheses were as follows:

**Hypothesis** **1** **(H1).** *Compared to the control group, the MBSR group will display a greater decrease in perceived stress.*

**Hypothesis** **2** **(H2).** *Compared to the control group, the MBSR group will display a greater increase in heart rate variability.*

**Hypothesis** **3** **(H3).** *Compared to the control group, the MBSR group will display a greater increase in positive affect.*

**Hypothesis** **4** **(H4).** *Compared to the control group, the MBSR group will display a greater decrease in negative affect.*

**Hypothesis** **5** **(H5).** *Compared to the control group, the MBSR group will display a greater increase in subjective wellbeing.*

## 2. Materials and Methods

### 2.1. Study Design and Participants

The current study employed a quasi-experimental, nonrandomized design. The participants of this study were residents in Cities N and U, Gyeonggi Province, South Korea, who were registered members of community mental health centers. The specific selection criteria for the participants were as follows: (1) having been diagnosed with schizophrenia by a psychiatrist, (2) having insight into their condition based on a score of 9 or less according to the Scale to Assess Unawareness of Mental Disorder (SUMD) [30], (3) having symptoms of moderate severity as shown by a score of less than 41 on the Brief Psychiatric Rating Scale (BPRS), (4) being between 19 and 64 years of age and not having changed their regimen of major psychotropic drugs that may affect physiological indicators during the study period, (5) not having taken part in a program similar to this study within the last 6 months, and (6) understanding the objectives of this study and agreeing to participate in this study in written form. The exclusion criteria were (1) having difficulty in verbal and non-verbal communication, (2) having been assessed with an absence of insight based on a score of 10 or more according to the SUMD, (3) having severe psychiatric symptoms as shown by a score of 41 or more on the BPRS, (4) having major changes in their regimen of psychiatric drugs during this program, and (5) having been diagnosed with personality disorders or substance-related and addiction disorders.

**Figure 1:**
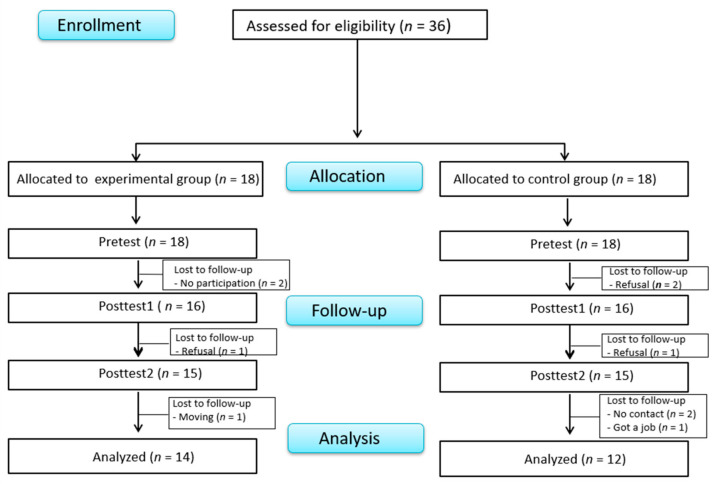
Flowchart of participants.

Power analysis (G*Power [31]) for a medium effect size (f2 = 0.25 [32], α = 0.05, B = 0.8) using a repeated-measures within-between interaction, with two groups, three points of measurement, and a correlation of 0.50 between repeated measures, indicated a minimum sample size of 28 participants. Considering a dropout rate of 20% based on a previous study [33], a total of 36 participants were recruited (18 participants with schizophrenia each for the experimental and control groups) among participants who expressed their intention to participate in the program and met the selection criteria (Figure 1). In the experimental group, two participants refused to further participate in this program after the first session, one participant participated in the program, but refused to fill out the questionnaire, and one participant moved to another place. In the control group, two participants discontinued participation for personal reasons, one participant refused to undergo HRV testing, two participants could not be reached afterward, and one participant discontinued participation for employment. The final sample size included a total of 26 participants, 14 in the experimental group and 12 in the control group.

### 2.2. MBSR Program

The MBSR program applied in this study was based on the principles articulated by Kabat-Zinn [23], and the researchers constructed the timing and content of the program to maintain the therapeutic environment of people with schizophrenia who visited the community mental health center. The MBSR program used in this study consisted of a total of eight sessions. To confirm its applicability to people with schizophrenia, the content and methods of the program were constructed with input from a professor of mind and body healing, who is an international expert in MBSR programs, and another professor of psychiatric and mental health nursing, who is a certified psychiatric mental health nurse. The principal investigator (PI) in this study is a certified psychiatric mental health nurse who has completed MBSR advanced training. The PI received MBSR training from an MBSR program expert who is the only certified leader in South Korea and who is recognized by the MBSR Headquarters (CFM: Center for Mindfulness) at the University of Massachusetts Medical School, USA. Furthermore, the PI’s doctoral dissertation was a study on an MBSR program for people with schizophrenia, and the PI currently educates university students about that program.

The theoretical rationale for the effect of the MBSR program, investigated in this study, is the self-regulation model according to which physical and mental health improves through mindfulness interventions [34,35]. According to this theory, the program was constructed in order to reduce stress in people with schizophrenia.

For the experimental group, the date and time of the program were decided in consideration of the schedule of the weekly rehabilitation program of the community mental health center through consultations with the center’s professionals. The content of each session of the program was as follows: session 1, orientation to the MBSR program and recognition of internal resources; session 2, perception and creative responding: a way to see and respond; session 3, pleasure and power in being present; session 4, how does conditioning and perception shape our experience; session 5, awareness of conditioned patterns of escape from difficulty and making selective reactions; session 6, awareness and balancing in stressful situations, especially acute or chronic stress; session 7, integrating mindfulness practice; session 8, keeping up mindfulness meditation in daily life and finishing the program. Participants were instructed to practice the meditation techniques that they had learned each week in the program regularly at home, to record notes on their practice in the provided booklet, and to share them with other participants when attending the following week’s session. The first author directed the program, and one social worker and one psychiatric nurse helped to implement the MBSR program. Following an MBSR program for people with an anxiety disorder [36], the program was carried out for eight weeks, with one session per week and 60 min per session (Table 1).

### 2.3. Measures

#### 2.3.1. Perceived Stress

Perceived stress was measured using a tool translated into Korean by Park and Seo [37] according to the Perceived Stress Scale (PSS) developed by Cohen [38]. This tool measures the extent to which an individual perceives their life over a month as unpredictable, uncontrollable, and excessively stressful. The tool has a total of 10 items on a five-point Likert scale from 0 (“strongly disagree”) to 4 (“strongly agree”). Items 4, 5, 7, and 8 are reverse-scored; thus, a higher average score corresponds to higher perceived stress. Regarding the internal consistency of the Korean version of the PSS, Cronbach’s α was 0.77 for negative perceptions and 0.74 for positive perceptions. This tool showed a good fit for measuring validity, and the criterion validity was determined to be high [37]. Cronbach’s α was 0.77 in this study.

#### 2.3.2. Heart Rate Variability (HRV)

HRV was measured using uBioMacpa Pro (BioSense Creative Co., Ltd., Seoul, Korea), which is a professional-grade HRV measurement instrument. Before starting the test in a quiet room, a participant sat in a chair for 5 min or more to reach calmness. Subsequently, a sensor measuring blood flow was attached to the skin surface of the index finger. This study measured short-term HRV for 2.5 min. A sensor was attached to the left index finger to measure the standard deviation of the normal-to-normal interval (SDNN). The SDNN is an antistress indicator. A SDNN value of 30 or more is considered to be normal, while lower values of the SDNN imply that an individual’s ability to adapt to stress is low and, simultaneously, that the degree of stress is high.

#### 2.3.3. The Positive and Negative Affect Schedule (PANAS)

Affect was measured using the Korean version of the Positive and Negative Affect Schedule (K-PANAS) by Lim [39], which is a translation and standardization of the Positive and Negative Affect Schedule (PANAS) developed by Watson et al. [40]. This tool has a total of 20 items on a five-point Likert scale from 1 (“not at all”) to 5 (“extremely”), consisting of 10 items regarding positive affect and 10 items regarding negative affect. Each score for positive and negative affect ranged from 10 to 50, with the higher scores indicating a higher level of affect. Regarding the reliability of this tool at the time of development in the research of Watson et al. [40], Cronbach’s α was 0.88 for positive affect and 0.85 for negative affect. Regarding the internal reliability of the K-PANAS [39] for mentally ill patients, Cronbach’s α was 0.87 for positive affect and 0.91 for negative affect. This tool showed favorable results for validity, and the criterion validity of this tool was high [39]. In this study, Cronbach’s α was 0.72 for positive affect and 0.70 for negative affect.

#### 2.3.4. Subjective Wellbeing

The subjective wellbeing of people with schizophrenia was measured using the Korean version of the Subjective Wellbeing under Neuroleptic Treatment Scale, Short Form (KvSWN-K), which was translated into Korean and validated by Kim et al. [41], according to the Subjective Wellbeing under Neuroleptic Treatment Scale (SWN) for people with schizophrenia developed by Naber et al. [42]. The KvSWN-K tool consists of five subfactors (emotional regulation, self-control, mental functioning, social integration, and physical functioning), and each subfactor consists of four items, totaling 20 items. This tool utilizes a six-point Likert scale from 1 (“not at all”) to 6 (“extremely”), and the score ranges from 20 to 120, with a higher score indicating higher subjective wellbeing. Ten items were reverse-scored. Regarding the internal reliability of this tool at the time of the development of KvSWN-K [41], Cronbach’s α was 0.87. This tool showed favorable validity, as well as high concurrent validity with the EuroQoL-5D. Cronbach’s alpha for each subscale ranged from 0.76 (social integration) to 0.88 (physical functioning) in the present study, and the overall Cronbach’s alpha was 0.92.

### 2.4. Data Collection

Before commencement of the study, the study objective and methods were explained to the directors of two community mental health centers located in G Province in South Korea, who provided permission and consent for program implementation. The data collection period was 11 December 2018 to 14 May 2019. To recruit participants, an information sheet on the MBSR program was distributed to people with schizophrenia in the community mental health centers. People with schizophrenia who were interested in participating in this program informed the psychiatric nurse who was in charge of the daytime rehabilitation program at the center. Regarding group allocation, two community mental health centers in Gyeonggi Province, which have similar service and treatment environments, were selected to prevent cross-contamination. All participants from one community mental health center were allocated to the experimental group and all participants from the other center were assigned to the control group. We selected participants with a similar distribution of gender, age, diagnosis, and symptoms in the two groups. After receiving consent from the center director, we met with patients who were eligible for participation at the center and explained the study objective and methods. Additionally, we promised to guarantee the anonymity and confidentiality of the collected data and to use the data for academic purposes only. Voluntary consent was then obtained verbally and in writing.

The experimental group was divided into two teams, each of which contained nine participants, for effective team dynamics and progression of sessions for people with schizophrenia. The program was conducted on Tuesdays and Thursdays. To minimize measurement errors between examiners, a standardized protocol was implemented. To avoid the halo effect during data collection, two research assistants (not the principal researcher) conducted the assessment sessions. All participants were assessed at three time points: pre-test (1 week before program implementation), post-test (immediately after the experimental treatment ended), and the follow-up test (6 weeks after the experimental treatment ended). After completing data collection, a gift was offered to participants as compensation. In line with a previous study [43] applying an MBSR program, this study conducted a follow-up test after 6 weeks. In the pre-test, the study objective was explained to the participants by two research assistants. Subsequently, we assessed participants’ general characteristics and evaluated perceived stress, HRV, positive and negative affect, and subjective wellbeing. Both the post-test and follow-up test followed a similar procedure, although the assessment of general characteristics was excluded. After completing data collection, the control group was informed in advance that the MBSR program would be provided twice in the future. After completion of the study, all participants in the control group who expressed interest were provided with two sessions of 60 min each.

### 2.5. Statistical Analysis

All data were analyzed using SPSS Statistics 20.0 for Windows (IBM Corp., Armonk, NY, USA). The frequencies, percentages, means, and standard deviations were calculated for participants’ general characteristics. Between-group differences were assessed using chi-square for categorical variables and independent *t*-tests for continuous variables (i.e., perceived stress, HRV, positive and negative affect, and subjective wellbeing). To analyze the effects of the MBSR program, a two-way repeated-measures ANOVA was used, with group as the between-group variable and assessment time as the within-group variable. Skewness and kurtosis tests were used to test the normality of the dependent variables. All variables were found to be normally distributed. Mauchly’s test of sphericity was carried out; if it yielded significant results, we used Wilks’ lambda for the multivariate tests, whereas, in the opposite case, we used the Greenhouse–Geisser test for univariate testing. Post hoc testing consisted of calculating changes in scores in the dependent variables from pre-testing, post-testing, and follow-up testing, followed by comparing these changes using the independent-sample *t*-tests.

### 2.6. Ethical Considerations

This study was approved by the Institutional Review Board (IRB) of K University (approval number: KHSIRB−18−041(NA)). All participants were informed about the content and purpose of this study and were provided a consent form to sign before the start of the study.

## 3. Results

### 3.1. Baseline Characteristics and Between-Group Differences

The experimental and control group were not statistically different according to the tested characteristic variables or baseline variables of interest (Table 2).

### 3.2. Hypothesis Testing

**Figure 2:**
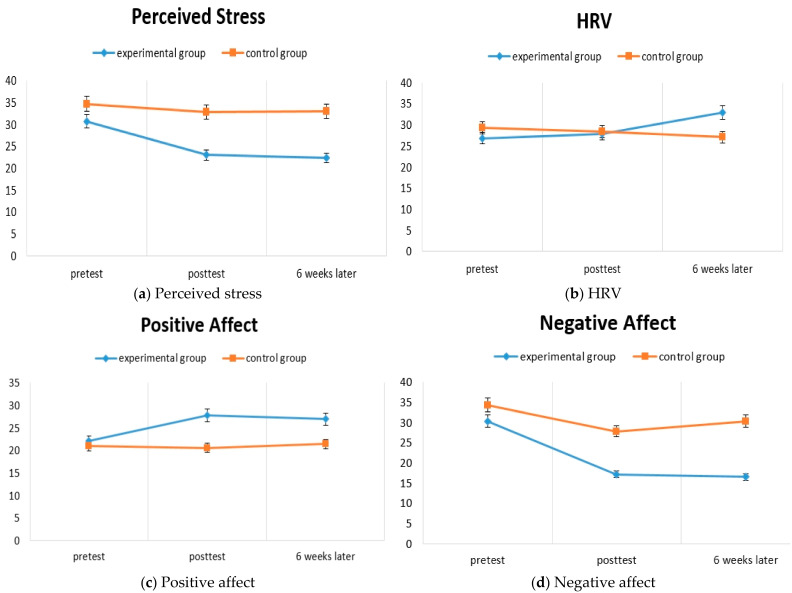

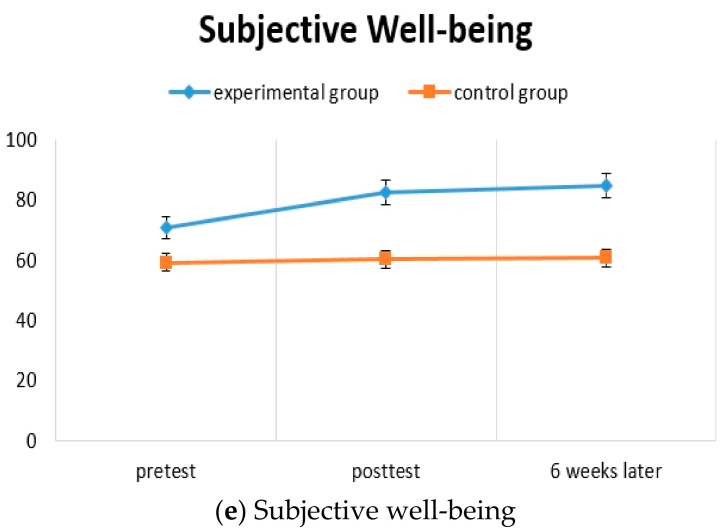
Hypothesis testing: (**a**) perceived stress; (**b**) HRV; (**c**) positive affect; (**d**) negative affect; (**e**) subjective wellbeing.

**Hypothesis 1** (“*Compared to the control group, the MBSR group will display a greater decrease in perceived stress.*”): Analyses revealed a significant group × time interaction (F(1.26, 30.26) = 3.95, *p* = 0.034, η^2^ = 0.256). Subsequent analyses showed a significant difference between groups at post-test (t(25) = −2.63, *p* = 0.015) and follow-up (t(25) = −2.87, *p* =0.008), such that the MBSR group scored lower on perceived stress relative to the control group (Figure 2a).

**Hypothesis 2** (“*Compared to the control group, the MBSR group will display a greater increase in heart rate variability*”): Analyses revealed a significant group × time interaction (F(1.57, 37.61) = 3.66, *p* = 0.042, η^2^ = 0.241). Subsequent analyses showed a non-significant difference between groups at post-test (t(25) = 0.40, *p* = 0.691) and follow-up (t(25) = 2.00, *p* = 0.057), such that the MBSR group scored higher on HRV relative to the control group (Figure 2b).

**Hypothesis 3** (“*Compared to the control group, the MBSR group will display a greater increase in positive affect*”): Analyses revealed no group × time interaction (F(1.17, 28.11) = 2.47, *p* = 0.107, η^2^ = 0.177). Although a significant effect for group was observed (F(1, 24) = 4.67, *p* = 0.045, η^2^ = 0.187), change over time was not statistically significant (F(1.17, 28.11) = 0.346, *p* = 0.346, η^2^ = 0.088) (Figure 2c).

**Hypothesis 4** (“*Compared to the control group, the MBSR group will display a greater decrease in negative affect*”): Analyses revealed a significant group × time interaction (F(2, 48) = 4.55, *p* = 0.022, η^2^ = 0.283). Subsequent analyses showed a nonsignificant difference between groups at post-test (t(25) = −1.87, *p* = 0.074) and a significant difference at follow-up (t (25) = −0.302, *p* = 0.006), such that the MBSR group scored lower on negative affect relative to the control group (Figure 2d).

**Hypothesis 5** (“*Compared to the control group, the MBSR group will display a greater increase in subjective well-being.*”): Analyses revealed no group × time interaction (F(1.33, 31.88) = 1.69, *p* = 0.207, η^2^ = 0.128). Although a significant effect for group was observed (F(1, 24) = 12.21, *p* = 0.002, η^2^ = 0.337), change over time was not statistically significant (F(1.33, 31.88) = 2.78, *p* = 0.083, η^2^ = 0.194) (Figure 2e). Appendix A Table A1 presents details on differences in dependent variables between groups over time.

## 4. Discussion

The experimental group, which received the MBSR program, showed decreases in perceived stress and negative affect, as well as significant improvements in HRV, compared to the control group. However, there were no improvements in positive affect or subjective wellbeing. These results are consistent with those of previous studies [43,44] that analyzed the effects of MBSR programs, and they indicate that the MBSR program had effects on perceived stress and HRV.

These results suggest that the eight-session MBSR program effectively reduced stress in patients with chronic schizophrenia residing in the local community. Although the standard MBSR sessions are commonly 150 min each, the currently program was modified, offering 60 min sessions due to the nature of the target sample. Indeed, persons with schizophrenia are more likely to have difficulty in concentrating [45], and the 60 min sessions were aligned with the programs offered at the community mental health centers. Despite the short duration of the sessions, this program was effective in reducing stress.

After conducting the program, HRV, which reflects a physiological response to stress, showed a significant increase in the experimental group compared to the control group. This result is consistent with that of a previous study [46,47], which observed changes in HRV after applying the breathing meditation method to healthy adults. These results can be attributed to activation of the parasympathetic nervous system and hypoactivation of the sympathetic nervous system through the MBSR program using meditation, which provides training in meditation with an attitude of nonjudgmental acceptance, along with awareness and intention. The results suggest that this program can be used as an effective method for improving adaptability to the external environment [47]. In addition, HRV reflects the activity of the vagus nerve, which is a major component of the parasympathetic nervous system. Therefore, a higher HRV implies mental and physical stability [48], and meditation leads to changes in the activation of the vagus nerve [49], affecting the ability to concentrate on sensory input and facilitating relaxation of mental and physiological tension. However, although a significant interaction was observed for HRV, post hoc analyses failed to reach statistical significance. As such, these results must be interpreted with caution.

There was no statistically significant difference in positive affect between the experimental and control groups. This result is inconsistent with that of a previous study [50] that observed a change in positive affect after patients with schizophrenia participated in meditation. An explanation for this inconsistency is that the meditation program provided for people with schizophrenia in the previous study [50] mainly consisted of loving–kindness meditation, which focuses on fostering feelings of being happy, cozy, and comfortable. Instead, the MBSR program applied in this study included various elements, such as breathing meditation, body scanning, yoga meditation, walking meditation, eating meditation, and loving–kindness meditation. Another possible reason is that the MBSR training in this study may have helped enhance awareness by connecting body and mind [23] rather than directly leading to changes in positive emotions such as happiness. Thus, this inconsistency could be attributed to negative symptoms such as anhedonia, apathy, and flat affect in people with chronic schizophrenia, which would offset any meaningful increase in positive affect. Therefore, additional research is required to investigate the effect of MBSR programs on positive affect.

Negative affect was reduced in the experimental group compared to the control group. This result is similar to those of previous studies that observed changes in negative affect in adults through meditation [51,52]. In addition, the results of this study are aligned with previous meta-analyses [24,25], which found that MBIs were effective for the negative symptoms of people with schizophrenia. An explanation for this result could be that the MBSR program allowed participants to choose an adaptive direction of cognition, departing from dysfunctional aspects through training to become aware of their thoughts and emotions [53]. In the post hoc analysis of negative affect, there was a significant difference between the experimental group and the control group at 6 weeks following the intervention, but not immediately after the intervention. A reason for this delayed effect might be that the researcher encouraged participants to engage in home practice and to continue mindfulness meditation by visiting the community mental health center once a week or having a phone consultation after the 8 week program. Furthermore, the delayed effect on negative affect in this study is consistent with a previous study [52] showing that perceived stress and negative affect gradually decreased in a linear fashion over time with sustained MBSR practice.

The MBSR program conducted in this study exhibited no effect on the subjective wellbeing of people with schizophrenia. In this aspect, the results of this study are inconsistent with those of a previous study [54] that observed a change in wellbeing after providing a meditation program for adults in the local community, as well as another study that applied an MBSR program to people with social anxiety disorder [55] that showed a difference. This inconsistency could be attributed to the distinct characteristics among participants, since the present study targeted patients with schizophrenia. The null association for subjective wellbeing may be attribute to baseline function. More specifically, the experimental group was marginally better off than the control group and may have experienced a ceiling effect, preventing significant change over time for this particular sample. Moreover, during the course of the study, the participants commented, “Mindfulness meditation gave me comfort,” “I felt lighthearted and relaxed,” “I felt agitated at the beginning, but, after patiently sticking with meditation, I felt at ease,” and “I became warmhearted and comfortable,” which all suggest that the participants’ wellbeing subjectively improved. Follow-up research is, therefore, required to investigate the effect of the MBSR program on subjective wellbeing in people with schizophrenia.

This study had some limitations. First, it is difficult to generalize the results of this study to all people with schizophrenia since participants were allocated to the experimental group and the control group by convenience sampling. Future research using randomized assignment is necessary. The participants of this study were limited to those who were assessed as having insight into their condition based on a score of 9 or less according to the SUMD [30], and those with good function, who were determined to have symptoms of moderate severity, with a score of 31 or more and less than 41 on the Brief Psychiatric Rating Scale (BPRS). Second, the participants who had relatively low levels of self-expression experienced difficulties in actively participating in the group because they exchanged relatively few verbal expressions while sharing meditation experiences after meditation practice. Thus, future research requires a strategy to better motivate self-expression by adding words or pictures as examples to the workbook to promote smooth self-expression. Third, the program was conducted in the program room due to the conditions of the center; this space was somewhat narrow, and the floor was uncomfortable for sitting. It would be ideal to create a more suitable environment for practicing yoga and walking meditation.

To this end, the current study showed that MBSR may minimize stress and negative affect among South Korean outpatients with schizophrenia. The results of this study are important as they dispute the misconception that mindfulness meditation is not helpful for people with schizophrenia. In addition, a meaningful aspect of the MBSR program is that it can be applied as an intervention across community mental health centers. Further, once patients learn the techniques, they are able to continue their practice.

## 5. Conclusions

The MBSR program decreased perceived stress and negative affect and increased heart rate variability in people with schizophrenia. This study provides guidance for further programs based on mindfulness. Furthermore, in light of these results, incorporating MBSR programs into routine psychosocial interventions at community mental health centers may contribute to improvements in the treatment process of people with schizophrenia.

## Figures and Tables

**Table 1:** Mindfulness-based stress reduction program of the study.

Session	Themes	Content of the Program	Time (min)
1	Orientation to the MBSR program and recognition of internal resources	1. MBSR program introduction	20
		2. Mindful breathing and body scan meditation	25
		3. Review and discussion	15
2	Perception and creative responding: a way to see and respond	1. Mindful breathing and body scan meditation	20
		2. Mindful eating meditation	20
		3. Review and discussion	20
3	Pleasure and power in being present	1. Mindful breathing meditation	20
		2. Mindful hatha yoga meditation	25
		3. Meditation discussion	15
4	How does conditioning and perception shape our experience	1. Mindful breathing and sound meditation	10
		2. Mindful walking meditation	30
		3. Review and discussion	20
5	Awareness of conditioned patterns of escape from difficulty and making selective reactions	1. Mindful breathing and mindful sound meditation	20
		2. Mindful thoughts and emotions meditation: review and discussion	20
		3. Explanation of stress response and mindfulness autonomic response	20
6	Awareness and balancing in stressful situations, especially acute or chronic stress	1. Mindful breathing meditation and mindful sound meditation	20
		2. Mindful thoughts and emotions meditation	20
		3. Review and discussion	20
7	Integrating mindfulness practice	1. Mindful breathing and body scan meditation	20
		2. Review and discussion	20
		3. Generosity in interpersonal relationships	20
8	Keeping up mindfulness meditation in daily life and finishing the program	1. Mindful breathing meditation	25
		2. Review and stress reduction discussion	25
		3. Explain home practice and finishing meditation	10

**Table 2:** Baseline characteristics and between-group differences (*N* = 26).

Characteristics	Categories	Exp. (*n* = 14)	Cont. (*n* = 12)	χ^2^/t	*p*
		*n* (%)/M ± SD	*n* (%)/M ± SD		
Gender	Male	8 (30.8)	7 (26.9)	0.01	1.000 ^†^
	Female	6 (23.1)	5 (19.2)		
Age (year)	28–59	46.43 ± 8.25	44.42 ± 7.03	0.66	0.514
Education	≤Middle school	2 (7.7)	4 (15.4)	2.06	0.725
	≥High school	12 (46.1)	8 (30.8)		
Marital status	Single	11 (42.3)	10 (38.5)	0.90	0.638
	Married	3 (11.5)	2 (7.7)		
Religion	Yes	9 (34.6)	6 (23.1)	1.37	0.713
	No	5 (19.2)	6 (23.1)		
Cohabitants	Family	12 (46.1)	9 (34.7)	0.70	0.952
	None	2 (7.7)	3 (11.5)		
Working status	Employed	3 (11.5)	1 (3.9)	0.85	0.598 ^†^
	Unemployed	11 (42.3)	11 (42.3)		
Monthly income	None	4 (15.4)	8 (30.8)	4.13	0.127
	Less than 850 USD	10 (38.4)	4 (15.4)		
Disease duration (years)	≤5	1 (3.8)	2 (7.6)	23.32	0.224
	6~9	2 (7.6)	1 (3.8)		
	≥10	11 (42.4)	9 (34.8)		
Number of psychiatric hospitalizations	No	1 (3.8)	2 (7.7)	2.39	0.792
	1–2	5 (19.2)	5 (19.2)		
	≤3	8 (30.9)	5 (19.2)		
Age at first onset	≤20	2 (7.6)	4 (15.2)	17.28	0.504
	21–29	7 (26.8)	2 (7.6)		
	30–39	5 (19.0)	6 (23.8)		
Presence of support groups	Yes	9 (34.6)	8 (30.8)	0.02	1.000 ^†^
	No	5 (19.2)	4 (15.4)		
Perceived stress		30.71 (6.56)	34.67 (4.23)	−1.79	0.086
HRV		26.82 (16.70)	29.34(15.43)	−0.40	0.695
Positive affect		22.14 (5.63)	21.00 (4.80)	0.56	0.582
Negative affect		30.36 (6.54)	34.33 (4.52)	−1.77	0.089
Subjective wellbeing		70.64 (17.43)	59.00 (9.69)	2.05	0.051

HRV = heart rate variability; Exp. = experimental group; Cont. = control group; ^†^ Fisher’s exact test; M ± SD = mean ± standard deviation.

## Data Availability

The data presented in this study are available from the authors upon reasonable request.

## Appendix A

**Table A1:** Differences in dependent variables between groups over time.

Variable	Group	Pre-Test	Posttest 1 (Post 8 Weeks)	Posttest 2 (Post 14 Weeks)	Source	F (df1, df2)	*p*	Differences (Post 1-Pre)			Differences (Post 2-Pre)		
		M ± SD	M ± SD	M ± SD				M ± SD	t (df)	*p*	M ± SD	t (df)	*p*
Perceived Stress	Exp.	30.71 ± 6.56	23.00 ± 5.17	22.36 ± 4.60	G	17.69 (1, 24)	<0.001	−7.71 ± 5.89	−2.63 (25)	0.015	−8.35 ± 5.60	−2.87 (25)	0.008
	Cont.	34.67 ± 4.23	32.83 ± 5.84	33.00 ± 7.23	T	9.17 (1.26, 30.26)	0.001	−1.84 ± 5.46			−1.67 ± 6.29		
					G × T	3.95 (1.26, 30.26)	0.034						
HRV	Exp.	26.82 ± 16.70	27.90 ± 12.69	32.98 ± 14.43	G	0.48 (1, 24)	0.848	1.08 ± 16.01	0.40 (25)	0.691	6.16 ± 12.39	2.00 (25)	0.057
	Cont.	29.34 ± 15.43	28.36 ± 12.05	27.13 ± 12.00	T	1.08 (1.57, 37.61)	0.356	−0.98 ± 8.42			−2.21 ± 8.04		
					G × T	3.66 (1.57, 37.61)	0.042						
Positive Affect	Exp.	22.14 ± 5.63	27.79 ± 9.42	27.00 ± 8.81	G	4.67 (1, 24)	0.045	5.65 ± 7.50	1.76 (25)	0.090	4.86 ± 7.56	1.19 (25)	0.246
	Cont.	21.00 ± 4.80	20.58 ± 8.96	21.41 ± 10.19	T	1.11 (1.17, 28.11)	0.346	−0.42 ± 10.00			0.41 ± 11.37		
					G × T	2.47 (1.17, 28.11)	0.107						
Negative Affect	Exp.	30.36 ± 6.54	17.21 ± 5.26	16.50 ± 4.45	G	2.91 (1, 24)	0.101	−13.15 ± 9.26	−1.87 (25)	0.074	−13.86 ± 7.60	−3.02 (25)	0.006
	Cont.	34.33 ± 4.52	27.83 ± 9.81	30.33 ± 8.64	T	24.35 (2, 48)	<0.001	−6.50 ± 8.77			−4.00 ± 9.08		
					G × T	4.55 (2, 48)	0.022						
Subjective Well-being	Exp.	70.64 ± 17.43	82.35 ± 16.82	84.71 ± 15.24	G	12.21 (1, 24)	0.002	11.71 ± 17.83	1.39 (25)	0.177	14.07 ± 16.56	1.81 (25)	0.083
	Cont.	59.00 ± 9.69	60.25 ± 19.97	60.66 ± 19.15	T	2.78 (1.33, 31.88)	0.083	1.25 ± 20.54			1.67 ± 18.37		
					G × T	1.69 (1.33, 31.88)	0.207						

Cont. = control group; Exp. = experimental group; M = mean; SD = standard deviation.; G = group; T = time; G × T = group × time; df = degree of freedom.
